# Cyclodextrins in Parkinson’s Disease

**DOI:** 10.3390/biom9010003

**Published:** 2018-12-21

**Authors:** Marisa C. F. Barros, Ana C. F. Ribeiro, Miguel A. Esteso

**Affiliations:** 1 Department of Analytical Chemistry, Physical Chemistry and Chemical Engineering, University of Alcalá, 28871 Alcalá de Henares, Madrid, Spain; miguel_esteso@hotmail.com; 2 Department of Chemistry, University of Coimbra, 3004-535 Coimbra, Portugal; anacfrib@ci.uc.pt

**Keywords:** Parkinson, levodopa, cyclodextrins, controlled drug delivery systems, transport properties, thermodynamic properties, density, partial molar volumes, viscosity, mutual diffusion coefficients

## Abstract

Parkinson’s disease is a movement disorder characterized by a progressive degeneration of dopaminergic neurons that has been object of study by the scientific community through the last decades. However, nowadays there is still no treatment to cure it, although there are drugs available, with limited efficacy, to relieve the symptoms or replenish the cells with dopamine to supply the lack of dopaminergic neurons. This work was structured in two parts. In the first one, binary aqueous solutions of l-dopa and cyclodextrins were studied. In the second part, ternary aqueous solutions of l-dopa were studied with each of the selected cyclodextrins. In all cases, thermodynamic properties (density, partial molar volume and thermodynamic transfer functions for temperatures between 294.15 ± 0.01 K and 312.15 ± 0.01 K) and transport properties (mutual diffusion coefficients, viscosity, transfer viscosity at 298.15 ± 0.01 K and 310.15 ± 0.01 K) were studied. Using theoretical models to adjust the experimental data obtained for the diffusion coefficients and for the apparent molar volumes, in the ternary aqueous solutions, it was possible to estimate the values to the l-dopa-cyclodextrin association constant. For the aqueous ternary solutes, the partial molar volume of transfer of levodopa in the presence of the cyclodextrins, the partial molar expansibility at infinite dilution and from this, the Hepler constant, were determined. Also, the values of Gibbs free energy (Δ*G*^0^), enthalpy (Δ*H*^0^) and entropy (Δ*S*^0^) were determined. From the obtained information, it was possible to characterize the molecular interactions, as well as to identify some structural characteristics of the controlled drug delivery systems under study and to estimate the influence of the cyclodextrin substituent groups, and, also, the temperature effect in the interaction levodopa-cyclodextrin. It is our intent to attain information about the mechanism of possible new systems for controlled drug delivery systems, throughout an alternative perspective, which could allow to increase its effectiveness in the Parkinson’s treatment.

## 1. Introduction

Parkinson disease (PD) is the most frequent movement disorder in the Western countries, after Alzheimer’s disease, which turn it into one of the most studied diseases since its discovery [1]. Its incidence in industrialized countries is close to 0.3% of the general population, affecting more men than women. It is estimated that there are 6.3 million in the world, according to a report of the Global Declaration for Parkinson’s Disease (2004). This disease can appear in the range from 20 to 80 years old, with a maximum incidence between 55 and 65 years old, presenting an exponential increase in prevalence, depending on age, of around 3% after 65 years old [1]. In the first 4–5 years, the pharmacological treatment for PD is mainly performed with a dopamine precursor, l-3,4-dihydroxyphenylalanine also known as levodopa or l-dopa [2,3,4,5,6,7].

When discovered, in the early of 1960’s l-dopa was one of the most surprising neurological drugs as a result of its ability to induce a direct improvement in patients with Parkinson’s disease, due to its conversion into dopamine in both the central nervous system and the peripheral nervous system [8].

Recently, some studies have provided new data on the mechanism of action of l-dopa as well as on the side effects induced by it. New forms of administration and therapeutic combinations with l-dopa have also recently emerged [9].

Levodopa has proven to be the best symptomatic treatment for PD and, at some point in the disease, all patients need to take it. However, its long-term efficacy is limited by complications, due to the continued use of medication, such as dyskinesia’s and tremors [10,11,12,13].

The controlled drug delivery systems differ from the usual in the fact that they allow the correct amount of the active pharmaceutical ingredients in the formulation to arrive to the exact place and at the right time. This type of system allows to attain a decrease in adverse effects and a longer activity time, keeping a constant concentration of the drug at the site of action. Also, in this way it is possible to use, without so many complications, drugs whose solubility in water is very low or those drugs whose therapeutic range is reduced and offer protection to those who are more sensitive to enzymatic attacks or degradation by pH (usual conditions in the intestinal tract).

Several studies have been conducted on the use of controlled drug delivery systems in the treatment of PD. Currently, the most commonly excipient used is hydroxypropyl methylcellulose, while cyclodextrins are not currently used in therapeutics of PD [14]. However, cyclodextrins (CD) are among the most important excipients and have proven to be very useful in the development of controlled drug delivery systems for, for example, local anaesthetic. These carbohydrates have the advantage of improving the physicochemical properties (such as water or membrane solubility) of the encapsulated drugs, pharmacodynamics (potentiation of the therapeutic effect), pharmacokinetics (control of absorption and distribution in tissues) and minimize toxic effects (less local and systemic toxicity).

With the aim of achieving a broad study, are presented results with cyclodextrins with different characteristics: a natural, β-CD and a derivative, hydroxyalkylated cyclodextrin, HP-β-CD. The β-CD (natural cyclodextrin with seven glucopyranose units) it is present in most of the existing formulations in the market, even though it is reduced solubility, which could limits its pharmaceutical application, it has a high capacity to form complexes with a large number of molecules, being used as excipient for a big number of pharmacological preparations. In addition to these important facts, it should be noted that β-CD is available in high quantities and at low prices. The HP-β-CD is a hydroxyalkylated cyclodextrin, which is commonly used to increase water solubility of the drug, maintaining their characteristics in solution and their bioavailability.

Each cyclodextrin has its own abilities to form inclusion complexes with specific molecules, which depend how the hydrophobic molecule fit in the cyclodextrin cavity. The formation of inclusion complexes is determined by the characteristics of the host molecule, such as its polarity, size and geometry, which must be appropriate to the hydrophobic characteristics and dimensions of the cyclodextrin cavity. The formation of complexes can occur by insertion, in the cavity, of the entire molecule of the drug or by the entry of only a part of it.

The interactions responsible for the formation and stabilization of complexes are the electrostatic ones, as Van der Waals forces, hydrophobic interactions and hydrogen bonds [15]. On the other hand, in aqueous solution the water molecules that fill the central cavity of the cyclodextrins are in an unfavourable energy situation due to the polar-nonpolar interactions. When complexes are formed, water molecules are substituted by drug molecules, which have a less polar character than water molecules. This process is energetically favourable in terms of enthalpy and entropy, which leads to a decrease in the total energy of the system [16].

In this work, it is carried out the study of thermodynamic and transport properties, in order to show possible advantages of the use of cyclodextrins in systems of controlled release of drugs, in the treatment of Parkinson’s disease.

Taking into account the main characteristics of the different cyclodextrins tested and, taking into consideration that the main drug used in the treatment of PD, l-dopa, has a limited solubility in water, which conditions its distribution by means of corporal fluids, as well as presents high adverse effects, it seems an advantageous therapeutic alternative its inclusion in a controlled drug release system with cyclodextrins.

In this way, it is discussed in the present work the possibility to improve the applicability of l-dopa, through the use of cyclodextrins to enable new formulations of the drug in the treatment against Parkinson’s disease.

## 2. Theoretical Aspects

### 2.1. Concepts of Diffusion

The diffusion is an irreversible phenomenon of great importance in nature. This can be observed in physical, chemical and biological processes that involve mass transfer [17].

Diffusion occurs when, in a solution, there is a concentration gradient, free of the effect of convection currents, which causes a spontaneous flow of matter that tends to reduce the above-mentioned difference in concentration and to restore, in this way, the balance of the system. In the absence of a concentration gradient, the particles of the solutes present in the solution are in permanent movement, known as Brownian motion, resulting in a system in equilibrium.

The quantification of the diffusion is done through the diffusion coefficient of each component in the solution, relating its flow with its concentration gradient. However, the driving force of the diffusion is, in thermodynamic rigor, the gradient of chemical potential of the diffusing substance which, for ideal solutions and at a constant temperature, coincides numerically with the value of the concentration gradient.

Therefore, we may contemplate the following approaches to define the isothermal diffusion: the thermodynamics of irreversible processes and Fick’s laws [18]. 

Mutual diffusion coefficient, *D*, in a binary system (system with two components), can be defined by the concentration gradient (without convection or migration) by the Fick’s first law:

(1) $$J=-D\frac{\partial c}{\partial x}$$

where *J* presents the flow of matter across a reference plane per time unit per area unit and, in a one-dimensional system and *c* is the concentration of solute, in moles per volume unit at the point considered; and Equation (1) may be used to measure *D*. The diffusion coefficient may also be measured considering Fick’s second law:

(2) $$\frac{\partial c}{\partial t}=\frac{\partial }{\partial x}\left(D\frac{\partial c}{\partial x}\right)$$

Generally, the experimental methods used, analyse the diffusion phenomenon by confining it to a one-dimensional process, due to the facility to accomplish the mathematical treatment at one-dimension, that is later generalized to a three-dimensional space. Considering *D* as a constant, the Equation (2), for a unidimensional process, becomes very easy to determine. This estimate can only be applicable when the differences of concentration are small, just like in the Taylor dispersion technique, experimental method used in this work [18,19]. 

In the case of ternary systems, Fick’s laws (Equations (1) and (2)) are insufficient to carry out an adequate analysis of their diffusion process, since the diffusive flow of each of the independent components can be affected by the magnitude of the concentration gradients of the remaining components present in the medium [19,20,21]. So, in this particular case diffusion is described by an extension of the Fick diffusion equations (Equations (3) and (4)),

(3) $$-\left(J_{1}\right)={\left(D_{11}\right)}_{v}\frac{\partial c_{1}}{\partial x}+{\left(D_{12}\right)}_{v}\frac{\partial c_{2}}{\partial x}$$

(4) $$-\left(J_{2}\right)={\left(D_{21}\right)}_{v}\frac{\partial c_{1}}{\partial x}+{\left(D_{22}\right)}_{v}\frac{\partial c_{2}}{\partial x}$$

where $J_{1},\;J_{2},\;\partial C_{1}/\partial x\;and\;\partial C_{2}/\partial x\;$ are the molar fluxes and the gradients in concentration of solute 1 and solute 2, correspondingly. Main diffusion coefficients, $D_{11}\;and\;D_{22}$, give the flux of each solute produced by its own concentration gradient. Cross diffusion coefficients, $D_{12}\;and\;D_{21}$, give the coupled flux of each solute driven by a concentration gradient in the other solute. A positive $D_{ik}$ cross-coefficient (i≠k) indicates a co-current coupled transport of solute *i* from regions of higher concentration of solute *k* to regions of lower concentration of solute *k*. However, a negative $D_{ik}$ coefficient indicates a counter-current coupled transport of solute *i* from regions of lower to higher concentration of solute *k*.

The knowledge of the intermolecular interactions that occur in the multicomponent diffusion processes can provide us with important information about what happens in the dissolution. Some authors have developed models that quantify these molecular interactions. For example, there is a theoretical model, developed by Paduano et al. [22,23,24,25,26], which has been used to perform the estimation of the values of the association constants in the case of multicomponent systems, in chemical equilibrium, involving cyclodextrins. Its theoretical basis and the equations that allow to correlate the diffusion coefficients with the equilibrium constants, are adequately described by these authors, so in this work the most relevant aspects that lead to the determination of the above-mentioned equilibrium constants.

### 2.2. Thermodynamic Properties of Aqueous Solutions

#### 2.2.1. Partial Molar Volume and Apparent Molar Volume

A detailed review of the concept of apparent and partial molar volumes of electrolytes and ions in solutions, initially presented by Millero in 1971 [27] is well described in literature [27].

The partial molar volume of a solute, $V^{0}$, is the limiting value of its partial molar volume when the solute concentration, *c*, approaches infinite dilution in the solvent. 

When the number of moles of solute approaches zero, that is, when the interactions between solute and solvent are barely produced in the aqueous solution, that is, in a situation of infinitesimal concentration, the partial molar volume of the solute, $V^{0}$, coincides numerically with its apparent molar volume, $\phi_{V}^{0}$.

The partial molar volume of the solute depends on the density of the solution at solute concentration, *m*, as:

(5) $$\emptyset_{V}=\frac{M_{2}}{\rho }+\frac{1000}{m}\frac{\rho_{1-}\rho }{\rho_{1}\rho }$$

where $M_{2}$ is the molar mass of the electrolyte solute and $\rho \;and\;\rho_{1}$ are respectively the densities of the solution and the pure solvent.

To obtain the values of the apparent partial molar volume, $\phi_{V}^{0}$, in a situation of infinitesimal concentration, for electrolytes in aqueous solution in this work, it is used Masson equation [28], based on the square root of the molar concentration of the solute in the solution:

(6) $$\emptyset_{V}=\;\phi_{V}^{0}+S_{V\;\;}^{0}\sqrt{c}$$

where $S_{V}^{0}$ is a parameter characteristic of each solute, which is obtained by adjusting the experimental data to this equation.

For the case of non-electrolyte solutions, the apparent partial molar volume of the solute was determined by the Redlich equation [29], that assumes a linear dependence with the molar concentration, according to the following equation:

(7) $$\emptyset_{V}=\;\phi_{V}^{0}+b_{V}c$$

in which *b_V_* represents the slope and is directly related to the solute-solute interactions that take place in the solution. 

The hydrophobicity of the solute can be evaluated from the value of slope of the apparent molar volume versus the solute concentration (Equation (7)). If that slope is positive, it means that the hydrophobic interaction predominates. 

In the present work were used solutions that contain more than one solute, as so the determination of thermodynamic properties becomes more complex. 

The study of electrolyte solutions in these conditions was largely studied by Young and Smith [30], who formulated their additivity rule, making possible the calculation of an “average” value for the apparent partial molar property under study, in a solution containing various solutes, as a sum of the apparent partial molar properties independent of each of the solutes present in the solution.

In this way the apparent partial molar volume of a solute in a multicomponent solution is given by:

(8) $$\emptyset_{V}=\frac{m_{2}\emptyset_{2}+\;m_{3}\emptyset_{3}}{m_{2}+m_{3}}$$

where the concentrations, *m* and the apparent partial molar volumes, $\phi_{V}^{}$, of the different solutes are referred to by the respective subscripts 2 and 3.

To analyse the behaviour of one of the solutes in a ternary solution (two solutes and a solvent), depending on the presence of the solvent and the other solute, it is possible to consider the two as one single species in solution (the solvent and the second solute), that is, as a mixed solvent. Thus, the analysis of the interactions of the solute under study (first solute), in the presence of this mixed solvent, is done by defining a transfer molar volume, $∆\phi_{V}^{0}$ which is the difference between the partial molar volumes (or partial apparent molar) at infinitesimal concentration, of the solute whose study is intended (first solute), in the mixed solvent considered (the solvent plus the other solute) and the partial molar volume (or apparent partial molar) in the pure solvent. In accordance with the above, we have:

(9) $$∆\phi_{V}^{0}=(\phi_{V}^{0}\;mixed\;solvent)-\;(\phi_{V}^{0}\;pure\;solvent)$$

In a situation of infinitesimal concentration, the interactions between the solute molecules are practically non-existent, which means that the observed transfer volumes are only the result of the interactions between the molecules of the solute and those of the solvent.

The changes in the volume that occurs in the molecules of a solute when it is transferred from water to a mixed solvent, is discussed on base on the Friedman and Krishnan model [31].

#### 2.2.2. Influence of Temperature on Partial Molar Volumes at Infinitesimal Concentration

The effect of temperature dependence on $\phi_{V}^{0}$ is studied by fitting the partial molar volumes to a polynomial of the following type in terms of absolute temperature *T*:

(10) $$V_{\phi }^{0}\;=a_{0}+a_{1}T+a_{2}T^{2}$$

where *T* is the temperature in Kelvin and range, *a*_0_, *a*_1_ and *a*_2_ coefficients are evaluated by least-square fitting of $\phi_{V}^{0}$.

The standard apparent molar expansibility can be obtained by differentiating the previous equation with respect to temperature as follow:

(11) $$E_{2}^{0}={\left[\frac{\partial \phi_{V}^{0}}{\partial T}\right]}_{P}$$

The limiting apparent molar expansibility, $E_{2}^{0}$, gives essential information regarding solute-solvent interactions in solutions [32,33].

The second derivative of the limiting apparent molar expansibility was defined by Hepler [33] as a thermodynamic criteria to define the making or breaking character of the particular solute when dissolved in a solvent. 

(12) $${\left[\frac{\partial E_{2}^{0}}{\partial T}\right]}_{P}={\left[\frac{\partial^{2}\phi_{V}^{0}}{\partial T^{2}}\right]}_{P}$$

If the sign of ${\left[\frac{\partial E_{2}^{0}}{\partial T}\right]}_{P}$ is positive, the solute is a structure maker (kosmotropic behaviour) or on the other hand if the sign is negative the solute is structure breaker (chaotropic behaviour).

#### 2.2.3. Calculation of the Constants of Association of the Solutes from Values of the Partial Molar Volumes

The equilibrium constant for the association between cyclodextrins and a drug could be determined using a model proposed by Terekhova et al. [34,35], through a theoretical model whose main consideration is Young’s rule for a 1:1 binding model between a cyclodextrin and a drug.

#### 2.2.4. Thermodynamic Properties of the Complexation Process

In a chemical transformation, the state functions, enthalpy, ∆*H*, entropy and ∆*S* and Gibbs free energy ∆*G* are related one to each other by the equation [36]:

(13) ∆G=∆H−T∆S

A chemical equilibrium situation can be described through the variation of Gibbs free energy, ∆*G*^0^ [36]:

(14) $$∆G^{0}=-RTlnK_{p}$$

where *R* presents the ideal gas constant, *T* the temperature and *K_P_* the equilibrium constant at constant pressure. This equation describes the position of the chemical equilibrium as a function of the free energy of the reactants and products of the chemical reaction, at a constant pressure of 1 atm. This equation is also very useful to understand the state of equilibrium. Obtaining the values of the cyclodextrin-drug equilibrium constants, as a function of temperature, it is possible to establish the direction in which the position of the equilibrium moves. This law was proposed by Van’t Hoff [36]:

(15) $${\left[\frac{\partial lnK_{P}}{\partial T}\right]}_{P}=\frac{∆H^{0}}{RT^{2}}$$

In this way it is possible to determine the values of the constant *K* from the values of $\phi_{V}^{0}$ for each of the components of the system and from the dependence of *K* on the temperature, to determine the thermodynamic functions $∆H^{0},\;∆S^{0}\;and\;∆G^{0}$, for the balance between the cyclodextrin and a drug.

### 2.3. Jones-Dole Coefficients and Viscosity of Aqueous Solutions

The equation that allows to relate the relative viscosity ($\eta_{r}=\frac{\;\eta }{\;\eta_{0}})$ of a solution of an electrolyte, with its concentration, *c*, was developed by Jones and Dole [37]:

(16) $$\frac{\eta }{\eta_{0}}=1+Ac^{1/2}+Bc+Dc^{2}$$

where η is the viscosity of the solution, η_0_ is the viscosity of the pure solvent and *A*, *B* and *D* are parameters coefficients dependents on the temperature. 

In this model, the coefficient *A* is related to the long-range intermolecular forces (solute-solute interactions) and can be an accurate aid to understand whether or not some kind of association occurs in the solution (however, for non-electrolytes in aqueous solution, this coefficient is usually very small and can even be insignificant [38,39,40]).

The Jones-Dole coefficient *B* is related to the solute-solvent interactions that take place in the solution and helps to evaluate the structure-making or structure-breaking character of the electrolyte in the solution. A coefficient *B* of positive viscosity is related to solutes with an organizing capacity of the structure of the solvent (structure-making). On the other hand, a negative coefficient *B* of viscosity is related to solutes that have the ability to break the structure of water (structure breaking).

In relation to the coefficient *D* of the previous equation, it is related to the solute-solute and solute-solvent interactions. This coefficient is significant as long as the electrolyte concentration in the solution is high. In the vast majority of studies carried out with electrolytes, the *D* coefficient is usually neglected, since these are commonly performed in the range of low solute concentrations [38].

## 3. Experimental Techniques

### 3.1. Materials and Solutions

Levodopa (3,4-dihydroxy-l-phenylalanine); (Sigma-Aldrich, St. Louis, MO, USA), CAS 59-92-7; purity ≥ 99%; molecular weight, MW = 197.19 g mol^−^^1^; β-cyclodextrin (Sigma, Kawasaki, Japan) C4767; CAS 7585-39-9; purity ≥ 97%; water content ≤ 14%; HP-β-cyclodextrin 2-hydroxypropyl-β-cyclodextrin, (Acros Organics, Illkirch Cedex, France) 297560250; CAS 128446-35-5; purity ≥ 97%; water content ≤ 7.5%. Solutions were prepared by direct weighing, with an accuracy of ±0.0001 g, both the solute and degassed Milli Q ultrapure water (κ = 6 × 10^−8^ S cm^−1^). The water content of the CD was taken into account in the concentration. 

### 3.2. Mutual Diffusion Measurements

Among experimental methods developed for measurements of mass diffusion coefficients in liquids, the Taylor dispersion technique has been used frequently for measuring binary diffusion coefficients of various solutions and lately applied for three-component systems [41,42,43,44,45,46,47,48,49,50]. The concepts and operation of this experimental method for the measurement of diffusion coefficients are well described in the literature so only the most relevant points are following highlighted.

In the Taylor dispersion technique, a small amount of a given solution is injected into a laminar carrier streams of solvent, or of solution at a different concentration, to flow throughout a long capillary tube. The length of the Teflon dispersion tube for this experimental assembly was measured directly by stretching the tube in a large hall and using two high quality theodolytes and appropriate mirrors to accurately focus on the tube ends. This technique gave a tube length of 3.2799 (±0.0001) × 10^4^ mm, in agreement with less-precise control measurements using a good-quality measuring tape. The radius of the tube, 0.5570 (±0.00003) mm, was calculated from the tube volume obtained by accurately weighing (resolution 0.1 mg) the tube when empty and when filled with distilled water of known density. 

In a pattern run, a sample of 0.063 mL of the solution under study (*c_j_* ± Δ*c*) is injected into the laminar carrier stream (*c_j_*) through a 6-port Teflon valve (Rheodyne, model 5020). The flow rate is keeping constant (0.17 mL min^−1^) with the assistance of a metering pump (Gilson model Miniplus 3) which allows retention times of about 1.1 × 10^4^ s. Both the dispersion tube and the injection valve are placed into an air thermostat bath to keep the temperature constant at 298.15 K ± 0.01 K.

The dispersion of the injected samples is monitored by using a differential refractometer (Waters model 2410) at the outlet of the dispersion tube. Voltage values as a function of the elapsed time, *V*(*t*), are measured at accurately 5 s intervals by using a digital voltmeter (Agilent 34401 A). Binary diffusion coefficients are calculated from the dispersion equation

(17) $$V\left(t\right)=V_{0}+V_{1}t+V_{max}{\left(t_{R}/t\right)}^{1/2}\exp[–12D{\left(t\;–\;t_{R}\right)}^{2}/r^{2}t]$$

being the additional fitting parameters: *t_R_*, the mean sample retention time; *V_max_*, the peak height; *V*_0_, the baseline voltage; and *V*_1_, the baseline slope.

Extensions of the Taylor technique have been used to determine ternary mutual diffusion coefficients (*D*_ik_) for multicomponent solutions. These *D*_ik_ coefficients are evaluated from the fitting of two or more replicate pairs of peaks for each carrier-stream, to the ternary dispersion equation

(18) $$V\left(t\right)=V_{0}+V_{1}t+V_{max}\;{\left(\frac{t_{R}}{t}\right)}^{\frac{1}{2}}\left[W_{1}\exp\left(\frac{–12D_{1}{\left(t\;–t_{R}\right)}^{2}}{r^{2}t}\right)+\left(1-W_{1}\right)\exp\left(\frac{–12D_{2}{\left(t\;–t_{R}\right)}^{2}}{r^{2}t}\right)\right]$$

The two pairs of refractive –index profiles, *D*_1_ and *D*_2_, are the eigenvalues of the matrix of the ternary *D*_ik_ coefficients. *W*_1_ and 1 –*W*_1_ are the normalized pre-exponential factors [48]. 

In standard experiments, small volumes, Δ*V*, of a solution of composition (*c*_1_ + ∆*c*_1_) and/or (*c*_2_ + ∆*c*_2_), are injected into carrier solutions of composition (*c*_1_, *c*_2_) at time *t* = 0 [17,41]. The mixtures in the results reported by our experimental method were prepared using mass fractions and then converted to molar concentration by means of the relation *w_i_ = c_i_(M_i_/ρ)* where *w_i_* stands for the concentration in mass fraction, *c_i_* is molar concentration, *M_i_* is the molar mass of the constituent *i* and *ρ* the density of the mixture. 

### 3.3. Density Measurements

The density of these solutions was obtained by using an Anton Paar DMA 5000M densimeter, with precision of 1 × 10^−6^ g cm^−3^ and accuracy of 5 × 10^−6^ g cm^−3^ in the ranges of 0−90 °C of temperature and 0–10 bars of pressure. This apparatus is provided with a Peltier system which allows keeping constant the temperature of the samples into the vibrating-tube within ±0.005 degrees. The measurements were carried out between 294.15 K and 312.15 K. The density value for each solution studied was the mean one of at least four sets of measurements. These mean values were reproducible with an uncertainty better than 0.001%.

### 3.4. Viscosity Measurements

The viscosity measurements were performed with an Ostwald type viscometer, calibrated from water [39,40]. These measurements were performed in a transparent-walled circulating water thermostat-bath, after immersing for a minimum of 2 h to achieve thermal equilibrium. The temperature was monitored at 298.15 ± 0.02 K and 310.15 ± 0.02 K, by using a digital thermometer. The efflux time was determined with a stopwatch with a resolution of 0.2 s. The arithmetic mean value from a minimum of four sets of flow times, for each solution, was taken to calculate such viscosity values. Because the efflux times were always in the range 350–400 s, no kinetic energy correction (Hagenbach correction) was applied. The viscosity values found were reproducible within ±0.1% of uncertainty (±0.001 mPa s).

## 4. Results and Discussion

In this chapter the results of the experimental diffusion coefficients, volumetric and viscosimetric techniques for some transport and thermodynamic properties of l-dopa in presence of cyclodextrins are presented. To a better understanding of the behaviour of l-dopa in presence of the cyclodextrins, the results obtained for binary aqueous solutions of l-dopa and these values are also presented and compared with the ternary systems. 

### 4.1. Binary Aqueous Systems

The experimental mutual diffusion coefficients of binary aqueous solutions of l-dopa at 298.15 K and 310.15 K are already published by Barros et al. [47].

Looking at the obtained results for the l-dopa, it is possible to observe that, at the lowest studied concentrations, the aqueous diffusion coefficient average presents a decrease with the solution’s concentration increase, in both studied temperatures. However, from the concentration (0.00375 mol dm^−3^) ahead, the diffusion coefficient presents a rise of approximately 6%, regardless the temperature. From the diffusion coefficient’s data, it was possible to calculate values of the activity coefficients. These values decrease as the solute concentration increases, although there is no significant variation with the temperature. This decrease might be related to a major increase of the solute-solvent interactions with the concentration’s increase, relatively to the solute-solute ones.

The hydrodynamic radius of the l-dopa molecule was calculated from the obtained values of the diffusion coefficient. It was found that the hydrodynamic radius of the l-dopa molecule reaches a maximum value of concentration rounding *c* = 2.50 × 10^−3^ (mol dm^−3^), in both temperatures, which leads to the conclusion that at higher concentrations, the viscosity effect upon the diffusion coefficient value is not significant. From the values of the hydrodynamic radius at infinitesimal concentration, at different concentrations of l-dopa, it was possible to determine the molar hydrodynamic volume that presents the values of 144 cm^3^ mol^−1^ at 298.15 K and 145 cm^3^ mol^−1^ at 310.15 K.

The density values of binary aqueous solutions of l-dopa determined in the temperature range of 294.15–312.15 K, as well as the partial molar volumes determined from the obtained values of the density, are presented in Table S1. They show a linear increase with the concentration’s increase for all studied temperatures. The density values at infinite dilution decrease as the temperature increases. This decrease might be a consequence of a volume increase caused by the thermal effect.

The partial molar volumes will be established from the obtained values of the density, which present an increase with the increasing concentration of the solute in the solution, at all temperatures. This might be related to the rise of the attractive solute-solvent interactions originated by the increase in solute concentration. This behaviour is very similar for all the studied temperatures, which indicates that temperature has no influence on the partial molar volume behaviour. The obtained values for the partial molar volumes, at infinitesimal concentration, point out that in l-dopa aqueous solutions, the interactions solute-solvent type should be predominant. These interactions increase with the temperature increase, which indicates that the solution is more structured at higher temperatures.

From the dependence of the l-dopa partial molar volumes, at infinitesimal concentration, on the temperature, it is possible to attain the value of the molar expansibility at infinite dilution, $E_{2}^{0}$. This presents a positive value, indicating the existence of strong solute-solvent interactions, which suggests that this solute has a structure-making behaviour.

The results of the viscosity of binary aqueous solutions of l-dopa at 298.15 K and 310.15 K are already published by Barros et al. [47]. It was observed that the viscosity values of the l-dopa aqueous solutions increase with the solute concentration and decrease with the temperature increasing. From this analysis, according to Jones-Dole equation, it is observed that the *A* coefficient value is very small and negative, for both studied temperatures, from which it is deduced the existence of very weak interactions of the solute-solute type, being them more significant at the temperature of 298.15 K. On the other hand, once the viscosity *B* coefficient value is positive, we may conclude the existence of strong solute-solvent interactions and also that l-dopa is a structure-making solute type, which is in accordance with the obtained results for the other studied properties.

The study of β-cyclodextrin and HP-β-cyclodextrin properties in aqueous solutions allowed verifying that these cyclodextrins present aspects of their behaviour quite similar between each other when they are in aqueous solutions.

The diffusion of these two cyclodextrins was previously studied by Santos et al. [51]. This author found that the diffusion coefficient values from β-CD and from HP-β-CD “*are small and little influenced by the increase in the concentration of the solution, regardless of what temperature is considered*.” It must be taken into account that cyclodextrins are very large molecules, with a high molar mass, so they present a high resistance to movement and, consequently, it is expected that they have a low diffusion coefficient.

Density values have been determined and, from them, the values of partial molar volumes of the solutions of the CDs under study, at different concentrations and in the temperature range between 294.15 K and 312.15 K. The obtained values for β-CD and for HP-β-CD are presented, in Tables S2 and S3, respectively.

From the obtained results it is possible to observe that the HP-β-CD presents higher values for density and apparent molar volumes, while the β-CD presents the smallest values. Looking at the studied cyclodextrins, the results suggest that the HP-β-CD is the one that most interacts with the water molecules, incorporating a relatively high number of them into its hydration sphere (simultaneously two phenomena occur: hydrophobic hydration—water molecules more or less immobilized in the surrounding area of the hydrophobic areas of the molecule—and hydrophilic hydration -water molecules anchored around the OH of the hydroxy-propyl- groups). 

Regarding the behaviour of the ϕ_V_ vs. the molality of the solutions, we can see that these slopes are quite close to each other, presenting values that are always higher than those of $\phi_{V}^{0}$, which suggests that in these aqueous solutions there is a predominance of solute-solute type interactions over the ones of the solute-solvent type.

The apparent partial molar volumes of β-CD and HP-β-CD present a linear adjustment as a function of the molality of the solution. 

From the dependence of the partial molar volumes of β-CD and HP-β-CD, at infinitesimal concentration, on the temperature, it is possible to attain the value of the molar expansibility at infinite dilution, $E_{2}^{0}$. For the studied cyclodextrins, the values of the molar expansibility at infinitesimal concentration $E_{2}^{0}$ were determined. They are positive values, which suggests that in aqueous solution these CDs present a structure-making behaviour. 

The viscosity values of the aqueous solutions of β-CD and HP-β-CD were previously determined by Santos et al. [51]. Those studies were made at different concentrations of the CD and at the temperatures of 298.15 K and 310.15 K, and they support the conclusion that both the cyclodextrins have a structure-making behaviour*,* in agreement with the behaviour observed by other authors, as well as in this work from other physicochemical properties analysed.

### 4.2. Ternary Aqueous Systems

**Figure 1:**
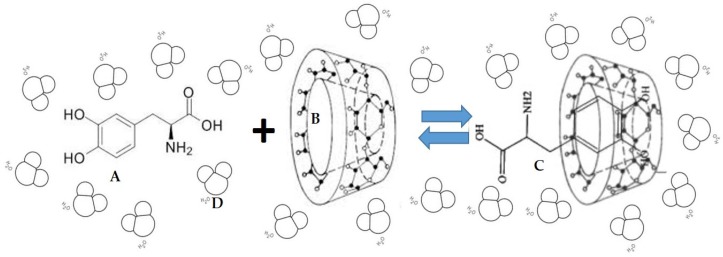
Schematic (speculative) representation of the formation of inclusion complexes l-dopa - β-CD (**C**), between a molecule of l-dopa (**A**) and another of β-CD (**B**) and (**D**) represents the water molecules.

It is very likely that interactions between molecules of l-dopa and CD molecule may occur. Assuming that, a 1:1 type complex can be formed between a molecule of l-dopa and a molecule of cyclodextrin, as drew in Figure 1.

The association process can be described by the equilibrium:

l − dopa + CD ↔ l − dopa.CD (19)

whose association constant, *K*, is:

(20) $$K=\frac{\left[Ldopa.CD\right]}{\left[Ldopa\right]\left[CD\right]}$$

Using theoretical models to adjust the experimental data obtained for the main and cross diffusion coefficients, it was possible to estimate values for the association constant K, of l-Dopa-cyclodextrin, considering the mixture water + cyclodextrin as a mixed solvent and the kind of complexes formed are always of the type 1:1.

The diffusion coefficients values of l-dopa in presence of cyclodextrins have already been published by the Barros et al. [48,49,50].

From the published experimental data values of the diffusion coefficients it was possible to estimate values for the constant K, as well as for the diffusion coefficients of the species in equilibrium, *D*_11_*, D_22_* and D_33_*, estimated for the aqueous solutions of levodopa in the presence of β-CD and HP-β-CD, at 298.15 K and 310.15 K. These values are presented in Tables S4 and S5, respectively.

Taking into account that the values for the association constants of Tables S4 and S5 were estimated by applying the Paduano et al. [22,23,24,25,26] theoretical model, in an attempt to understand what occurs in the solutions, from the comparison of the obtained results for the different cyclodextrins under study, it can be deduced that between the l-dopa and the corresponding cyclodextrin (β-CD and the HP-β-CD) complexation may occur. Moreover, for the β-CD the values suggest that this complexation is strongest at the lowest temperature, 298.15 K, while for the HP-β-CD this interaction increases drastically when the temperature increases to 310.15 K.

From the application of the theoretical model of Paduano et al. [22,23,24,25,26] to the diffusion measurements, it was possible to estimate the diffusion coefficient values of the associated species, D_33_*, for the same systems.

For the β-CD case it is observed that D_33_* presents a value close to that of D_22_*, mainly at 298.15 K. This result allows to state that the l-dopa molecule can be totally, or partially, included in the cyclodextrin cavity and so, the dimensions of the diffusing species, cyclodextrin or complex l-dopa-cyclodextrin, are very similar, especially for the lowest temperature, 298.15 K, where the inclusion is stronger, which is in accordance with the value determined for the association constant.

For the HP-β-CD case, D_33_* presents a different value from that of D_22_*, for the two studied temperatures. This result seems to indicate that the estimated value for K may be mainly due to the existence of external interactions between the molecules of l-dopa and HP-β-CD, than to the inclusion of l-dopa in the cyclodextrin cavity.

The obtained values of the apparent partial molar volumes point to the idea that there is an association between l-Dopa and cyclodextrins β-CD and HP-β-CD. In addition, it is observed that l-dopa in the presence of β-CD presents values of the association constant higher than in the presence of HP-β-CD, which is in line with what has been concluded through the diffusion coefficient values. Finally, it is found that for β-CD the association constant, K, decreases with increasing temperature and that for the HP-β-CD, K, it increases with increasing temperature.

In this work, the experimental values of the density of aqueous solutions of l-dopa in the presence of different cyclodextrins, at different temperatures are presented. From them, the corresponding values of the apparent molar volumes were determined, using Equation (5) and considering the mixture water + cyclodextrin as a solvent. From the obtained values, the interactions between the solute, the solvent and the co-solvent, are discussed.

The experimental values of the density, together with those calculated for the corresponding apparent molar volumes, for aqueous solutions of l-dopa in presence of β-CD and HP-β-CD, in the temperature range between 294.15 K and 312.15 K, are presented in Tables S6 and S7, respectively. In all cases, the standard deviations of the density measurements were less than 5.0 × 10^−6^, which leads to an uncertainty associated with the calculation of the apparent molar volumes, always lower than 5%. The concentration range selected for the solutions of l-dopa, between 0.5–7.5 mol kg^−1^, was chosen not to exceed the limit of solubility of this drug.

Analysing the density values of these aqueous ternary solutions of l-dopa in the presence of the β-CD and HP-β-CD, it is observed that they present density values higher than those of the corresponding binary aqueous solutions, for all the concentrations and temperatures studied. It is also observed that as the temperature increases, the density of the solutions decreases, although always maintaining a parallel behaviour for the different temperatures studied. In all cases, the results obtained responded to linear adjustment, with values of density of the solvent, *ρ*_0_, very close to those of the density of the binary aqueous solutions of the β-CD or HP-β-CD, with the same molal concentration.

In relation to the apparent molar volumes, ϕ_V_, calculated from the values of the density, it should be noted that they are always positive and that, in general, they have higher values than those of the l-dopa in aqueous solution. That is, in general, the presence of cyclodextrins in the medium causes an increase in the value of the apparent molar volume of l-dopa. 

The values of the apparent molar volumes at infinitesimal concentration can be determined from the Equation (8), whose values coincide with those of the partial molar volumes), $\phi_{V}^{0}$ = $V_{2}^{0}$. These values are presented in Tables S7 and S8.

Comparing these values with those obtained for the binary systems: water + β-CD/water + HP-β-CD and water + l-dopa, at infinitesimal concentration, the values corresponding to their partial molar volumes of transfer are obtained [according to the Equation (9).

Thus, Tables S8 and S9 present the calculated values for the partial molar volumes of the transfer of the l-dopa from water to the mixed solvent water + β-CD and water + HP-β-CD at the different concentrations studied, in the range of temperatures between 294.15–312.15 K.

The different behaviour of l-dopa in relation to the complexation processes with the studied cyclodextrins, can be analysed through the changes of the transfer volumes. As can be observed at Table S8, in the presence of β-CD the l-dopa shows, for almost all concentrations and temperatures studied, positive volumes of transfer increasing with the concentration and the temperature. This indicates that the interaction l-dopa-β-CD decreases as both the variables get higher. Therefore, it is highly probable that the release of the l-dopa molecules, which may be initially included in the cavity of the β-CD, occurs in parallel with the conformational changes that take place with the increase in temperature. By applying the Friedman and Krishnan model to this system [31], we can conclude that the hydrophilic-ionic type and/or of the hydrophilic-hydrophilic type are the predominant interactions.

Looking at the volumes of transfer of l-dopa in the presence of HP-β-CD we checked that they have negative values for almost all temperatures and concentrations. In addition, in this system the partial molar volumes of transfer decrease with the increase in concentration, which can be interpreted, mainly, as a sign of the formation of inclusion complexes l-dopa-HP-β-CD. Thus, applying the model of Friedman and Krishnan [31], it can be stated that the predominant interactions that take place in this system could be either hydrophilic-hydrophobic or between hydrophobic groups.

Taking into account the analogy between the structures of the cited two cyclodextrins to explain the volume changes that accompany the transfer of l-dopa from water to water + cyclodextrin, it is evident that the volume variations determined for each different media are totally different, verifying that the inclusion is favoured by the presence of the hydroxypropyl groups, instead of the hydroxyl groups.

To examine how the addition of l-dopa affects the structure of the mixed solvents, the partial molar expansibility at infinite dilution can also be used and, from this, it is possible to determine the Hepler constant for the systems under study. 

The values of partial molar expansibility at infinite dilution for the l-dopa in presence of different concentrations of β-CD and HP-β-CD are presented in Tables S10 and S11.

Since a positive value of $E_{2}^{0}$ indicates the existence of strong solute-solvent interactions and that a negative value of $E_{2}^{0}$ shows that the solute-solvent interactions are very weak, l-dopa acts as a structure-breaking solute of the mixed solvents water-β-CD and water-HP-β-CD, once the partial molar expansibility at infinitesimal concentration always presents positive values. 

The second derivative of the partial molar volume at infinite dilution was a criterion of hydrophobicity proposed by Hepler [33], according to:
if$E_{2}^{0}=\left[\frac{\partial V_{\phi }^{0}}{\partial T}\right]>\;0_{P}$y$\left(\frac{\partial E_{2}^{0}}{\partial T}\right)<\;0$, the solute is considered hydrophilicif$E_{2}^{0}=\left[\frac{\partial V_{\phi }^{0}}{\partial T}\right]<\;0_{P}$y$\left(\frac{\partial E_{2}^{0}}{\partial T}\right)>\;0$, the solute is considered hydrophobic

These values are presented in Table S12.

Through the obtained results for these systems it can be deduced that the l-dopa, in the presence of the mixed solvents water + β-CD and water + HP-β-CD, presents hydrophilic characteristics. On the contrary, for the mixed solvent water + NaSO_3_-β-CD, the l-dopa presents negative values of $E_{2}^{0}$, demonstrating the presence of very weak, or almost non-existent, solute-solvent interactions. 

It is possible to estimate the values of the constant *K* from the values of $\phi_{V}^{0}$ for each of the components of the system and from the dependence of *K* on the temperature, to determine the thermodynamic functions ∆*H*^0^, ∆*S*^0^ and ∆*G*^0^ for the balance between the cyclodextrin and a drug. These values are presented in Tables S13 and S14.

The calculated values of ∆*G*^0^ for the systems under study show that the process of complexation between l-dopa and β-CD and l-dopa and HP-β-CD is spontaneous (∆*G*^0^ < 0).

Usually, the inclusion of a drug in the cyclodextrin cavity is associated to negative values of ∆*H*^0^ and negative or slightly positive values of ∆*S*^0^, showing that the inclusion process is not accompanied by desolvation but it is a process governed mainly by the enthalpy [24]. The present estimation shows that only the complex formed between l-dopa and β-CD presents this behaviour, with an enthalpy term predominating in the formation of the inclusion complex.

The inclusion of l-dopa in HP-β-CD presents a different result. In fact, when the l-dopa is dissolved in the solution, it seems to have strong interactions with the solvent. Once the inclusion complex is formed, an unfavourable entropy change occurs due to the rupture of the solvation sphere, which explains a positive value of ∆*S*^0^, indicating that the formation of the inclusion complex with HP-β-CD is governed by the entropy term.

The experimental values obtained for the viscosity of the solutions of l-dopa considering the mixed solvent mixture water + β-CD and mixture water + HP-β-CD were determined and are presented in Tables S15 and S16. In all cases, the standard deviation of these viscosity measurements was less than 3.5 × 10^−4^ units.

To determine the variation of the viscosity of these solutions, the solutions of l-dopa, at different concentrations, were prepared in each of the mixed solvents studied and which were formed by water and the corresponding cyclodextrin, at a constant concentration. This way, changes in the viscosity of these solutions are, consequently, attributed only to the l-dopa dissolved in the mixed solvent and not to the composition of the solvent.

From the analysis of the Tables S15 and S16 it can be seen that, as expected, the values obtained for the viscosity of the solutions of l-Dopa in the mixed solvent water + HP-β-CD and water + β-CD, increase with the increase of the concentration of the solute and decrease with temperature.

The analysis of these experimental values was made by adjusting them to the Jones-Dole equation (16) [37]. 

The values obtained for the coefficients A and B of the Jones-Dole equation, corresponding to the aqueous solutions of l-dopa in the presence of β-CD and HP-β-CD, are presented in Tables S17 and S18, for the studied temperatures of 298.15 K and 312.15 K.

As it can be seen from the observation of the values in Table S17, obtained for coefficient *A*, at 298.15 K and 310.15 K, for l-dopa in presence of β-CD, are small but they increase with the increase of the amount of β-CD in the dissolution. These data reveal that the solute-solute interactions are very weak but they increase with the increase of the amount of β-CD present in the solution.

For the system l-dopa in presence of HP-β-CD as it can be appreciated in Table S18, the values obtained for the coefficient *A* at 298.15 K are small and, in general, tend to decrease with increasing the amount of cyclodextrin present in the solution. On the other hand, at 310.15 K, it is seen that the value of *A* parameter increases with the increase of the concentration of cyclodextrin in the solution. This behaviour seems to indicate the existence of weak solute-solute interactions and that, in both cases, such interaction becomes more and more important with the increase of the amount of cyclodextrin in solution.

Analysing the viscosity coefficient *B* values for the l-dopa (Table S17), it is verified that, in general, they are positive but decrease with the increase of the concentration of the β-CD in the solution. From such behaviour it can be concluded that there are strong solute-co-solvent interactions that weaken as the amount of cyclodextrin present in the solution increases. Taking into account that the coefficient *B* provides information on the interactions that take place between the molecules of the solute and those of the solvent (in this case, of a mixed solvent), which result in a greater or lesser structuring of the solution (structure effect making or structure effect breaking, of the solute) [38], it is deduced that the l-dopa presents a behaviour of the structure breaking type at both temperatures, once the interactions decrease with the amount of β-CD present in the dissolution.

Analysing the values of the coefficient *B* for the l-dopa in presence of HP-β-CD, it is noticed that these are high and positive, although they do not present a definite variation with the increase of the cyclodextrin concentration in the solution. On the other hand, it is observed that the magnitude of the coefficient *B* is much superior than that of the coefficient *A*, suggesting that in this system there are weak solute-solute interactions and strong solute-solvent interactions. From the above, it is possible to conclude that l-dopa presents structure-making characteristics in the of HP-β-CD solutions.

Relatively to the viscosity *B*-coefficients of transfer, Δ*B*, for aqueous solutions of l-dopa in the presence of the cyclodextrins, it is possible to observe that the values of Δ*B* are, in general, positive for almost all the systems and studied temperatures. These results, together with those obtained for the viscosity measurements, show that the l-dopa presents structure maker characteristics in the presence of β-CD and HP-β-CD. This agrees with the results obtained from the partial molar expansibility at infinitesimal concentration and the Hepler constant.

## 5. Conclusions

This work had as principal purpose to find out how to improve the applicability of levodopa (l-dopa), using cyclodextrins, in a controlled drug delivery system, in order to contribute to the scientific community with a new possible formulation of this drug, used in Parkinson’s disease. For that, some possible formulations of the controlled drug delivery system in aqueous solution were proposed, each one containing l-dopa and a cyclodextrin and some of their physicochemical properties were characterized. This way, we intend to obtain useful information concerning the molecular interactions that occur in these systems, which can be applied in pharmaceutical research.

Levodopa was chosen as the active form, since it is the drug most used in the Parkinson’s disease treatment. Two different cyclodextrins, with different characteristics, were chosen to act as a vehicle. The first one was the simplest, the β-cyclodextrin (β-CD) and the other two were cyclodextrins derived from the previous one: a hydroxyalkylated, the HP-β-CD. This choice allowed us to carry out an extensive study, evaluating the effect of the substituent groups on the solubility of the drug (l-dopa), as well as to analyse and obtain relevant information on their interactions with this drug.

The behaviour of the aqueous solutions of l-dopa in the presence of the three cyclodextrins was studied, evaluating mutual diffusion coefficients, volumetric and viscosimetric properties.

From the properties of the aqueous solutions studied, it was verified that the interactions between the l-dopa and each of the cyclodextrins used are different. Using different theoretical models to adjust the experimental data obtained in the different techniques, it was possible to estimate the values of the l-dopa-cyclodextrin association constant, at various temperatures, always considering that we were in the presence of a mixed solvent formed by the water + cyclodextrin mixture and that the complexes that could be formed would be of the 1:1 type.

Regarding the estimated values for the association constant, K, from the experimental diffusion coefficients, we verified the possible existence of l-dopa complexation for l-dopa with β-CD and for l-dopa with HP-β-CD, and, on the other hand, for β-CD this association is stronger at the lower temperature, whereas, in the case of the HP-β-CD this interaction is superior at higher temperature. The diffusion coefficient values of the associated species are different for the different cyclodextrins studied. For β-CD, the values are very close to the main diffusion coefficient, especially at the temperature of 298.15 K, which shows that the l-dopa molecule can be totally or partially included in the cyclodextrin cavity. For HP-β-CD, the diffusion coefficient of the associated species is different from the main diffusion coefficient for the two studied temperatures, which may be due to the presence of external interactions between the l-dopa molecules and the HP-β-CD. Thus, it is concluded that there is a strong probability that the release of the l-dopa molecules, which may be initially included in the β-CD cavity, occurs at the same time as the conformational changes occur as a consequence of the increase in temperature. In the other hand for HP-β-CD interactions with l-dopa may even occur in the exterior of the cavity of that cyclodextrin.

The study of the apparent partial molar volumes reinforces the previous conclusions. That is, there is an association between l-dopa and cyclodextrins β-CD and HP-β-CD. Thus, it can be concluded that in the process of complexing l-dopa with the different cyclodextrins, the association constant, K, decreases with increasing temperature for β-CD and increases with increasing temperature for HP-β-CD.

From the study of the partial molar transfer volumes it is concluded that the predominant interactions are of the hydrophilic-ionic type and/or of the hydrophilic-hydrophilic type for the l-dopa-β-CD system; in the case of l-dopa-HP-β-CD, the hydrophilic-hydrophobic type or between hydrophobic groups are the predominant ones. Therefore, due to the fact that the variations in the volume determined in both mixed media are totally different and taking into account the analogy between the structures of the three cyclodextrins, it can be concluded that the inclusion of l-dopa in the cavity of the cyclodextrin is favoured by the presence of the hydroxypropyl groups and, to a lesser extent, by the hydroxyl groups.

For examining how the addition of l-dopa affects the structure of mixed solvents water + cyclodextrin, the partial molar expansibility at infinite dilution and, thereafter, the Hepler constant, were calculated.

It was concluded that l-dopa in the presence of the mixed solvent water + β-CD and water + HP-β-CD presents a structure-making behaviour, also exhibiting hydrophilic characteristics in the presence of these two solvents. 

The determination of the changes in Gibbs free energy allows evaluating the spontaneity of the thermodynamic processes. Thus, from the values of Δ*G*^0^ obtained for the three systems studied, it was found that the complex between l-dopa and cyclodextrin is a spontaneous process (Δ*G*^0^ < 0) with the two studied cyclodextrins.

Taking into account that the inclusion of the guest into a host molecule, like a cyclodextrin, is a process mainly governed by enthalpy inclusion existent in the cyclodextrin cavity, such inclusion is associated with negative values of Δ*H*^0^ and at negative or slightly positive values of Δ*S*^0^. On this basis, the behaviour of the complex between l-dopa and cyclodextrins has been analysed, finding that only the complex formed between the l-dopa and β-CD presents this type of behaviour, with a predominant enthalpic term in the formation of the inclusion complex.

When l-dopa is dissolved in the solution, it appears to have strong interactions with HP-β-CD, which decrease as long as the formation of the complex between l-dopa and HP-β-CD progresses, due to the fact that the formation of these inclusion complexes is governed by the entropy term.

The values obtained of the viscosity study, as well as those of all the derived quantities, reinforce the previous conclusions.

In summary, it can be stated that there is an association between l-dopa with β-CD and l-dopa with HP-β-CD. The values of the association constant, estimated through measurements of the apparent partial molar volumes and the diffusion coefficient ones, as well as the obtained viscosity results, for both aqueous systems, are concordant with each other. However, it should be accepted that the values obtained for these association constants are only estimates to try to understand what happens in the solutions. In fact, depending on the experimental method used, values of different association constants are obtained, although of the same order of magnitude.

On the other hand, it is observed that with increasing temperature the association between β-CD and l-dopa decreases, which is indicative that the molecule of l- dopa may be encapsulated within the cyclodextrin, being released gradually as the temperature increases (towards the physiological temperature value). Given the possible pharmacological applications for these compounds (l-dopa and cyclodextrins) it can be concluded that the l-dopa + β-CD system presents a good possibility of being applied as a controlled drug delivery system in the Parkinson’s disease treatment.

## Supplementary Materials

Table S1. Densities, *ρ* and partial molar volumes, V_ϕ_, of l-Dopa aqueous solutions, at different concentrations, *m*, in the temperature range T = (294.15–312.15) K; Table S2. Densities, *ρ* and partial molar volumes, V_ϕ_, of β-CD aqueous solutions, at different concentrations, *m*, in the temperature range T = (294.15–312.15) K; Table S3. Densities, *ρ* and partial molar volumes, V_ϕ_, of HP-β-CD aqueous solutions, at different concentrations, Table S4. Values of the association constant, K and of the diffusion coefficients of the species in equilibrium, *D_11_**, *D_22_** and *D_33_**, estimated for the aqueous solutions of levodopa in the presence of β-CD, at 298.15 K and 310.15 K; Table S5. Values of the association constant, K and of the diffusion coefficients of the species in equilibrium, *D_11_**, *D_22_** and *D_33_**, estimated for the aqueous solutions of levodopa in the presence of HP-β-CD, at 298.15 K and 310.15 K; Table S6. Densities, *ρ* and partial molar volumes, V_ϕ_, of l-dopa in presence of β-CD aqueous solutions, at different concentrations, *m*, in the temperature range T = (294.15–312.15) K; Table S7. Densities, *ρ* and partial molar volumes, V_ϕ_, of l-dopa in presence of HP-β-CD aqueous solutions, at different concentrations, *m*, in the temperature range T = (294.15–312.15) K; Table S8. Partial molar volumes at infinitesimal concentration, $V_{\phi }^{0}$ = $V_{2}^{0}$ and partial molar volumes of transfer, $\Delta V_{\phi }^{0}$, for aqueous solutions of l-dopa in the presence of β-CD, at temperatures from 294.15 K to 312.15 K; Table S9. Partial molar volumes at infinitesimal concentration, $V_{\phi }^{0}$ = $V_{2}^{0}$ and partial molar volumes of transfer, $\Delta V_{\phi }^{0}$, for aqueous solutions of l-dopa in the presence of HP-β-CD, at temperatures from 294.15 K to 312.15 K; Table S10. Partial molar expansibility at infinite dilution, $E_{2}^{0}$, of l-dopa in presence, of β-CD at different concentrations; Table S11. Partial molar expansibility at infinite dilution, $E_{2}^{0}$, of l-dopa in presence, of HP-β-CD at different concentrations; Table S12. Hepler constant to the system l-dopa-β-CD and l-dopa– HP-β-CD; Table S13. Estimated thermodynamic properties for the complexation process of l-dopa with β-CD, at temperatures from 294.15 K to 312.15 K; Table S14. Estimated thermodynamic properties for the complexation process of l-dopa with HP-β-CD, at temperatures from 294.15 K to 312.15 K; Table S15. Viscosity of the solutions of l-dopa in presence of the mixed solvent water + β-CD, at 298.15 K and 310.15 K; Table S16. Viscosity of the solutions of l-dopa in presence of the mixed solvent water + HP-β-CD, at 298.15 K and 310.15 K; Table S17 Coefficients *A* and *B* of Jones-Dole equation and viscosity *B*-coefficients of transfer, Δ*B*, for aqueous solutions of l-dopa in the presence of β-CD, at 298.15 K and 310.15 K; Table S18. Coefficients *A* and *B* of Jones-Dole equation and viscosity *B*-coefficients of transfer, Δ*B*, for aqueous solutions of l-dopa in the presence of HP-β-CD, at 298.15 K and 310.15 K.
