# Supplementary material for: Cyclodextrins in Parkinson’s Disease
Source: Biomolecules. 2018 Dec 21;9(1):3. doi: 10.3390/biom9010003 (PMC6359444; doi:10.3390/biom9010003)
Supplement: Supplementary file 1 [file biomolecules-09-00003-s001.pdf]

Table S1. Densities, ρ, and partial molar volumes, V_ϕ, of L-dopa aqueous solutions, at different concentrations, *m*, in the temperature range T = (294.15-312.15) K

10^3 *m* /(mol Kg^{-1})	ρ /(g cm^{-3})	10^6 σ (a)	V_ϕ /(cm^3 mol^{-1})	ρ /(g cm^{-3})	10^6 σ (a)	V_ϕ /(cm^3 mol^{-1})
	**294.15 K**			**296.15 K**		
0(b)	0.997995	-	-	0.997541	-	-
0.243038	0.998013	1.4	122.60	0.997559	16.5	122.83
0.597434	0.998039	0.7	122.85	0.997585	1.4	122.99
1.06490	0.998074	0.7	123.05	0.997620	0.5	123.16
1.74325	0.998124	2.1	123.27	0.997670	2.1	123.35
2.45375	0.998176	20.5	123.48	0.997722	4.9	123.52
4.96705	0.998359	14.8	124.00	0.997905	3.5	123.99
6.99796	0.998505	15.6	124.35	0.998052	15.6	124.25
	**298.15 K**			**300.15 K**		
0(b)	0.997048	-	-	0.996517	-	-
0.243038	0.997066	3.5	123.27	0.996535	2.1	123.79
0.597434	0.997092	1.4	123.45	0.996561	1.4	123.98
1.06490	0.997127	0.7	123.61	0.996595	2.1	124.11
1.74325	0.997176	2.1	123.81	0.996644	2.8	124.30
2.45375	0.997228	4.2	123.96	0.996696	4.9	124.48
4.96705	0.997411	2.8	124.31	0.996877	2.8	124.89
6.99796	0.997557	15.6	124.52	0.997022	14.1	125.12
	**302.15 K**			**304.15 K**		
0(b)	0.995949	-	-	0.995345	-	-
0.243038	0.995967	4.2	124.44	0.995363	2.1	124.81
0.597434	0.995992	1.4	124.65	0.995388	0.7	124.98
1.06490	0.996026	0.7	124.86	0.995422	0.0	125.16
1.74325	0.996075	2.1	125.05	0.995471	2.1	125.34
2.45375	0.996126	4.9	125.21	0.995522	4.2	125.48
4.96705	0.996305	2.1	125.71	0.995700	2.1	125.93
6.99796	0.996448	14.8	125.98	0.995843	14.1	126.23

$10^3\ m$ /(mol Kg^{-1})	ρ /(g cm^{-3})	$10^6\ \sigma$ [(a)]	V_ϕ /(cm^3 mol^{-1})	ρ /(g cm^{-3})	$10^6\ \sigma$ [(a)]	V_ϕ /(cm^3 mol^{-1})
		**306.15 K**			**308.15 K**	
0[(b)]	0.994707	-	-	0.994036	-	-
0.243038	0.994725	1.4	125.26	0.994053	1.4	125.92
0.597434	0.994750	0.7	125.41	0.994079	0.8	126.12
1.06490	0.994784	0.7	125.61	0.994112	1.4	126.33
1.74325	0.994832	2.1	125.75	0.994160	2.1	126.54
2.45375	0.994883	4.9	125.89	0.994210	5.7	126.71
4.96705	0.995060	1.4	126.29	0.994385	0.4	127.18
6.99796	0.995203	13.4	126.54	0.994526	12.7	127.45
		**310.15 K**			**312.15 K**	
0[(b)]	0.993333	-	-	0.992599	-	-
0.243038	0.993350	0.7	126.29	0.992616	0.7	126.62
0.597434	0.993375	1.4	126.46	0.992641	0.7	126.79
1.06490	0.993409	2.1	126.60	0.992674	0.8	126.98
1.74325	0.993456	2.8	126.76	0.992722	2.1	127.15
2.45375	0.993506	4.9	126.87	0.992772	3.5	127.29
4.96705	0.993682	0.7	127.26	0.992946	0.6	127.71
6.99796	0.993823	13.4	127.50	0.993086	12.0	127.96

(a) σ represents the standard deviation of the measurements
(b) [47]

Table S2. Densities, ρ, and partial molar volumes, V_ϕ, of β-CD aqueous solutions, at different concentrations, *m*, in the temperature range *T* = (294.15-312.15) K

10^3 *m* /(mol Kg^{-1})	ρ /(g cm^{-3})	10^6 σ (a)	V_ϕ /(cm^3 mol^{-1})	ρ /(g cm^{-3})	10^6 σ (a)	V_ϕ /(cm^3 mol^{-1})
		**294.15 K**			**296.15 K**	
0 (b)	0.997995	-	-	0.997541	-	-
0.544374	0.998216	0.8	729.50	0.997762	0.4	730.56
2.48972	0.998994	0.2	733.66	0.998540	0.8	733.81
4.97590	0.999960	0.1	739.31	0.999510	0.6	738.66
7.43507	1.000890	0.5	744.16	1.000440	0.8	743.78
10.0000	1.001829	0.6	749.44	1.001385	0.9	748.60
		**298.15 K**			**300.15 K**	
0 (b)	0.997048	-	-	0.996517	-	-
0.544374	0.997268	0.5	731.09	0.996737	0.4	731.64
2.48972	0.998044	0.7	735.39	0.997513	0.3	735.36
4.97590	0.999008	0.6	740.65	0.998485	0.8	739.21
7.43507	0.999935	0.2	745.58	0.999421	0.7	743.46
10.0000	1.000868	0.1	751.20	1.000375	0.5	747.55
		**302.15 K**			**304.15 K**	
0 (b)	0.995949	-	-	0.995345	-	-
0.544374	0.996169	0.6	732.56	0.995565	0.6	733.13
2.48972	0.996943	0.7	736.36	0.996337	0.1	737.38
4.97590	0.997909	0.4	741.03	0.997301	0.4	742.05
7.43507	0.998839	0.8	745.57	0.998229	0.6	746.60
10.0000	0.999785	0.2	749.98	0.999170	0.3	751.31
		**306.15 K**			**308.15 K**	
0 (b)	0.994707	-	-	0.994036	-	-
0.544374	0.994926	0.5	734.27	0.994255	0.7	735.20
2.48972	0.995697	0.7	738.41	0.995027	0.5	738.23
4.97590	0.996655	0.2	743.90	0.995991	0.2	742.71
7.43507	0.997579	0.6	748.46	0.996919	0.8	747.20
10.0000	0.998510	0.7	753.78	0.997860	0.5	751.89
		**310.15 K**			**312.15 K**	
0 (b)	0.993333	-	-	0.992599	-	-
0.544374	0.993551	0.7	735.84	0.992817	0.7	736.64
2.48972	0.994323	0.7	739.07	0.993585	0.5	740.75
4.97590	0.995282	0.7	744.17	0.994545	0.8	745.04
7.43507	0.996210	0.6	748.27	0.995469	0.4	749.49
10.0000	0.997150	0.3	752.86	0.996401	0.6	754.71

(a) σ represents the standard deviation of the measurements
(b) [47]

Table S3. Densities, ρ, and partial molar volumes, V_ϕ, of HP-β-CD aqueous solutions, at different concentrations, m, in the temperature range T = (294.15-312.15) K

10^3 m /(mol Kg^{-1})	ρ /(g cm^{-3})	10^6 σ [(a)]	V_ϕ /(cm^3 mol^{-1})	ρ /(g cm^{-3})	10^6 σ [(a)]	V_ϕ /(cm^3 mol^{-1})
		**294.15 K**			**296.15 K**	
0[(b)]	0.997995	-	-	0.997541	-	-
0.500000	0.998225	1.2	919.91	0.997771	0.9	921.52
2.50144	0.999138	0.7	922.94	0.998681	4.9	924.36
4.96387	1.000242	0.7	926.30	0.999781	0.6	927.83
7.48862	1.001349	1.6	929.97	1.000885	1.8	931.60
10.0022	1.002427	1.3	933.74	1.001960	0.9	935.24
		**298.15 K**			**300.15 K**	
0[(b)]	0.997048	-	-	0.996517	-	-
0.500000	0.997277	0.7	922.55	0.996746	1.4	924.01
2.50144	0.998186	0.7	925.39	0.997652	0.1	926.85
4.96387	0.999284	0.2	928.88	0.998747	0.9	930.35
7.48862	1.000386	1.6	932.58	0.999847	1.8	933.98
10.0022	1.001459	0.1	936.29	1.000916	0.2	937.76
		**302.15 K**			**304.15 K**	
0[(b)]	0.995949	-	-	0.995345	-	-
0.500000	0.996177	0.7	925.08	0.995573	0.7	925.97
2.50144	0.997082	0.7	927.80	0.996476	0.1	928.86
4.96387	0.998175	0.7	931.44	0.997568	0.1	932.34
7.48862	0.999273	1.8	935.00	0.998663	1.6	936.11
10.0022	1.000341	1.6	938.79	0.999732	0.3	939.60
		**306.15 K**			**308.15 K**	
0[(b)]	0.994707	-	-	0.994036	-	-
0.500000	0.994935	1.4	927.28	0.994263	2.8	928.40
2.50144	0.995836	0.7	930.01	0.995164	1.4	931.06
4.96387	0.996925	0.2	933.60	0.996251	0.1	934.70
7.48862	0.998020	1.6	937.16	0.997342	1.8	938.40
10.0022	0.999082	0.7	941.09	0.998406	1.4	942.03
		**310.15 K**			**312.15 K**	
0[(b)]	0.993333	-	-	0.992599	-	-
0.500000	0.993560	2.1	929.54	0.992825	0.7	931.11
2.50144	0.994458	2.1	932.29	0.993721	0.0	933.98
4.96387	0.995543	0.3	935.97	0.994803	0.1	937.55
7.48862	0.996633	1.5	939.54	0.995890	1.4	941.12
10.0022	0.997694	0.1	943.30	0.996947	0.6	944.90

(a) σ represents the standard deviation of the measurements
(b) [47]

Table S4. Values of the association constant, K, and of the diffusion coefficients of the species in equilibrium, D_{11} *, D_{22} * and D_{33} *, estimated for the aqueous solutions of L-dopa in the presence of β-CD, at 298.15 K and 310.15 K.

C_1 /(mol·dm^{-3})	D_{11}^* /10^{-9} (m^2·s^{-1})	D_{22}^* /10^{-9} (m^2·s^{-1})	D_{33}^* /10^{-9} (m^2·s^{-1})	K /(dm^3·mol^{-1})
		**T=298.15 K**		
0.0005	0.560			
0.0015	0.557	0.324	0.320	60
0.0020	0.550			
		**T=310.15 K**		
0.003	0.809			
0.005	0.812	0.436	0.650	50
0.007	0.815			

Note: the data presented are those determined in the presence of a constant concentration of β-CD = 5.0 mM. D_{33} * represents the diffusion coefficient of the complex species, {L-dopa-β-CD}.

Table S5. Values of the association constant, K, and of the diffusion coefficients of the species in equilibrium, D_{11} *, D_{22} * and D_{33} *, estimated for the aqueous solutions of Levodopa in the presence of HP-β-CD, at 298.15 K and 310.15 K.

C_1 /(mol·dm^{-3})	D_{11}^* /10^{-9} (m^2·s^{-1})	D_{22}^* /10^{-9} (m^2·s^{-1})	D_{33}^* /10^{-9} (m^2·s^{-1})	K /(dm^3·mol^{-1})
		**T=298.15 K**		
0.0005	0.560			
0.0015	0.557	0.314	0.272	71
0.0020	0.550			
		**T=310.15 K**		
0.003	0.809			
0.005	0.812	0.405	0.551	190
0.007	0.815			

Note: the data presented are those determined in the presence of a constant concentration of H-β-CD = 5.0 mM. D_{33} * represents the diffusion coefficient of the complex species, {L-dopa-HP-β-CD}.

Table S6. Densities, ρ, and partial molar volumes, V_ϕ, of L-dopa in presence of β-CD aqueous solutions, at different concentrations, *m*, in the temperature range T = (294.15-312.15) K

$10^3\ m_{\text{L-Dopa}}$ /(mol Kg^{-1})	$10^3\ m_{\beta\text{-CD}}$ /(mol Kg^{-1})	ρ /(g cm^{-3})	V_ϕ /(cm^3 mol^{-1})	ρ /(g cm^{-3})	V_ϕ /(cm^3 mol^{-1})
		294.15 K		296.15 K	
0.520000	0.499980	0.998265	117.04	0.997807	121.94
1.550000	0.499980	0.998342	120.67	0.997878	126.21
3.041948	0.499980	0.998444	124.70	0.997971	130.51
5.122107	0.499980	0.998575	128.59	0.998085	135.31
7.165244	0.499980	0.998695	131.41	0.998190	138.39
0.497001	2.479425	0.999017	116.35	0.998560	123.66
1.521035	2.479425	0.999096	118.85	0.998632	125.87
3.053917	2.479425	0.999209	121.18	0.998734	128.30
5.021402	2.479425	0.999348	123.27	0.998859	130.41
7.456409	2.479425	0.999510	125.67	0.999007	132.37
0.527446	4.993389	1.000048	122.29	0.999595	127.00
1.651840	4.993389	1.000127	125.74	0.999668	130.59
3.063624	4.993389	1.000218	128.78	0.999754	133.21
5.041076	4.993389	1.000337	131.98	0.999865	136.28
7.014787	4.993389	1.000445	134.90	0.999969	138.57
0.537525	7.410455	1.000885	131.34	1.000434	135.64
1.601455	7.410455	1.000951	133.51	1.000496	137.78
3.040403	7.410455	1.001036	135.64	1.000575	139.88
4.959436	7.410455	1.001143	137.83	1.000676	141.75
6.942657	7.410455	1.001248	139.61	1.000775	143.29
0.527430	10.000811	1.001884	139.92	1.001438	141.44
1.490858	10.000811	1.001936	142.07	1.001489	143.50
2.952377	10.000811	1.002011	143.85	1.001560	145.77
4.946662	10.000811	1.002107	145.85	1.001652	147.82
6.957784	10.000811	1.002199	147.38	1.001739	149.52
		298.15 K		300.15 K	
0.520000	0.499980	0.997312	129.35	0.996773	133.19
1.550000	0.499980	0.997376	133.26	0.996833	137.17
3.041948	0.499980	0.997460	137.10	0.996910	141.43
5.122107	0.499980	0.997564	141.28	0.997005	145.64
7.165244	0.499980	0.997654	144.72	0.997090	148.56
0.497001	2.479425	0.998062	129.04	0.997531	131.61
1.521035	2.479425	0.998128	131.35	0.997595	133.60
3.053917	2.479425	0.998223	133.31	0.997687	135.60
5.021402	2.479425	0.998339	135.27	0.997799	137.46
7.456409	2.479425	0.998475	137.28	0.997930	139.44
0.527446	4.993389	0.999090	132.28	0.998569	136.90
1.651840	4.993389	0.999158	135.33	0.998633	139.39
3.063624	4.993389	0.999237	138.01	0.998707	141.85
5.041076	4.993389	0.999340	140.84	0.998805	144.15
7.014787	4.993389	0.999436	143.01	0.998895	146.26
0.537525	7.410455	0.999925	138.57	0.999413	142.52

10^3 m_{L-Dopa} /(mol Kg^{-1})	10^3 $m_{\beta\text{-CD}}$ /(mol Kg^{-1})	ρ /(g cm^{-3})	V_ϕ /(cm^3 mol^{-1})	ρ /(g cm^{-3})	V_ϕ /(cm^3 mol^{-1})
1.601455	7.410455	0.999984	140.67	0.999468	144.52
3.040403	7.410455	1.000060	142.41	0.999538	146.44
4.959436	7.410455	1.000155	144.43	0.999625	148.54
6.942657	7.410455	1.000250	145.80	0.999710	150.19
0.527430	10.000811	1.000923	144.00	1.000422	146.85
1.490858	10.000811	1.000971	145.98	1.000468	148.83
2.952377	10.000811	1.001040	147.96	1.000532	150.88
4.946662	10.000811	1.001127	150.16	1.000613	153.14
6.957784	10.000811	1.001210	151.77	1.000690	154.77
		**302.15 K**		**304.15 K**	
0.520000	0.499980	0.996205	136.79	0.995600	139.67
1.550000	0.499980	0.996261	141.01	0.995653	143.97
3.041948	0.499980	0.996332	145.40	0.995720	148.26
5.122107	0.499980	0.996418	149.80	0.995800	152.71
7.165244	0.499980	0.996494	152.82	0.995870	155.77
0.497001	2.479425	0.996959	134.77	0.996351	138.93
1.521035	2.479425	0.997020	136.64	0.996408	140.88
3.053917	2.479425	0.997107	138.66	0.996489	142.77
5.021402	2.479425	0.997214	140.36	0.996586	144.90
7.456409	2.479425	0.997340	142.08	0.996700	146.78
0.527446	4.993389	0.997990	140.65	0.997380	143.79
1.651840	4.993389	0.998049	143.54	0.997435	146.89
3.063624	4.993389	0.998119	145.65	0.997499	149.29
5.041076	4.993389	0.998208	148.18	0.997581	151.91
7.014787	4.993389	0.998293	149.89	0.997656	154.03
0.537525	7.410455	0.998830	144.29	0.998218	146.11
1.601455	7.410455	0.998883	146.47	0.998269	148.31
3.040403	7.410455	0.998950	148.58	0.998334	150.14
4.959436	7.410455	0.999033	150.58	0.998415	152.07
6.942657	7.410455	0.999115	152.10	0.998495	153.48
0.527430	10.000811	0.999832	149.54	0.999218	150.57
1.490858	10.000811	0.999875	151.70	0.999260	152.91
2.952377	10.000811	0.999935	153.89	0.999318	155.18
4.946662	10.000811	1.000010	156.28	0.999391	157.30
6.957784	10.000811	1.000080	157.96	0.999459	159.07
			**306.15 K**		**308.15 K**
0.520000	0.499980	0.994960	145.33	0.994285	149.50
1.550000	0.499980	0.995008	149.17	0.994329	153.22
3.041948	0.499980	0.995068	153.27	0.994384	157.04
5.122107	0.499980	0.995139	157.49	0.994449	160.95
7.165244	0.499980	0.995200	160.48	0.994500	164.39
0.497001	2.479425	0.995707	143.99	0.995036	150.17
1.521035	2.479425	0.995758	146.55	0.995079	153.60
3.053917	2.479425	0.995829	148.93	0.995137	156.78
5.021402	2.479425	0.995914	151.08	0.995200	160.31
7.456409	2.479425	0.996009	153.54	0.995269	163.31
0.527446	4.993389	0.996731	146.37	0.996066	150.89
1.651840	4.993389	0.996783	149.59	0.996114	153.70
3.063624	4.993389	0.996843	152.13	0.996169	156.16

$10^3\ m_{L\text{-}Dopa}$ /(mol Kg^{-1})	$10^3\ m_{\beta\text{-CD}}$ /(mol Kg^{-1})	ρ /(g cm^{-3})	V_ϕ /(cm^3 mol^{-1})	ρ /(g cm^{-3})	V_ϕ /(cm^3 mol^{-1})
5.041076	4.993389	0.996920	154.68	0.996237	158.95
7.014787	4.993389	0.996988	157.03	0.996299	160.93
0.537525	7.410455	0.997564	148.96	0.996905	151.73
1.601455	7.410455	0.997612	151.19	0.996950	154.06
3.040403	7.410455	0.997673	153.18	0.997006	156.21
4.959436	7.410455	0.997747	155.28	0.997074	158.49
6.942657	7.410455	0.997820	156.81	0.997140	160.14
0.527430	10.000811	0.998559	151.78	0.997907	153.39
1.490858	10.000811	0.998600	154.06	0.997946	155.69
2.952377	10.000811	0.998656	156.35	0.998000	157.89
4.946662	10.000811	0.998725	158.92	0.998068	160.12
6.957784	10.000811	0.998790	160.68	0.998130	161.96
		**310.15 K**			**312.15 K**
0.520000	0.499980	0.993579	152.74	0.992845	155.72
1.550000	0.499980	0.993620	156.32	0.992883	159.21
3.041948	0.499980	0.993670	160.33	0.992930	162.85
5.122107	0.499980	0.993730	163.93	0.992987	166.07
7.165244	0.499980	0.993780	166.70	0.993030	169.25
0.497001	2.479425	0.994328	153.59	0.993590	159.70
1.521035	2.479425	0.994367	157.76	0.993621	165.15
3.053917	2.479425	0.994417	161.54	0.993654	170.75
5.021402	2.479425	0.994470	165.26	0.993681	176.15
7.456409	2.479425	0.994520	169.26	0.993700	180.85
0.527446	4.993389	0.995357	153.29	0.994616	156.42
1.651840	4.993389	0.995402	156.29	0.994658	159.39
3.063624	4.993389	0.995452	159.08	0.994704	162.11
5.041076	4.993389	0.995515	161.76	0.994762	164.58
7.014787	4.993389	0.995569	164.20	0.994812	166.83
0.537525	7.410455	0.996194	154.54	0.995450	157.32
1.601455	7.410455	0.996236	156.94	0.995489	159.82
3.040403	7.410455	0.996288	159.10	0.995536	162.19
4.959436	7.410455	0.996350	161.52	0.995593	164.55
6.942657	7.410455	0.996410	163.20	0.995645	166.56
0.527430	10.00081	0.997194	155.34	0.996447	157.66
1.490858	10.00081	0.997232	157.44	0.996482	160.01
2.952377	10.00081	0.997283	159.84	0.996530	162.21
4.946662	10.00081	0.997346	162.30	0.996588	164.77
6.957784	10.00081	0.997405	164.00	0.996640	166.80

Table S7. Densities, ρ, and partial molar volumes, V_ϕ, of L-Dopa in presence of HP-β-CD aqueous solutions, at different concentrations, *m*, in the temperature range T = (294.15-312.15) K

10^3 m_{L-dopa} /(mol Kg^{-1})	10^3 $m_{HP-β-CD}$ /(mol Kg^{-1})	ρ /(g cm^{-3})	V_ϕ /(cm^3 mol^{-1})	ρ /(g cm^{-3})	V_ϕ /(cm^3 mol^{-1})
		**294.15 K**		**296.15 K**	
0.476397	0.500080	0.998295	120.19	0.997837	120.55
1.51641	0.500080	0.998373	121.87	0.997914	121.93
3.03180	0.500080	0.998485	122.76	0.998025	123.14
4.97062	0.500080	0.998624	123.84	0.998163	124.32
7.03538	0.500080	0.998769	124.70	0.998306	125.31
0.496817	2.50144	0.999132	123.66	0.998671	124.14
1.47082	2.50144	0.999202	124.37	0.998741	124.82
3.00163	2.50144	0.999312	125.07	0.998850	125.57
5.05041	2.50144	0.999456	125.82	0.998993	126.31
6.92625	2.50144	0.999586	126.38	0.999122	126.87
0.496884	4.96387	1.000186	117.65	0.999721	119.37
1.47060	4.96387	1.000262	118.62	0.999796	119.93
3.08245	4.96387	1.000384	119.97	0.999918	120.85
5.02990	4.96387	1.000528	121.19	1.000063	121.56
7.10753	4.96387	1.000682	121.70	1.000215	122.25
0.516978	7.48862	1.001393	116.78	1.000928	117.45
1.55137	7.48862	1.001473	118.88	1.001007	119.70
3.03198	7.48862	1.001582	121.12	1.001115	121.71
4.99891	7.48862	1.001722	122.98	1.001253	123.76
7.11888	7.48862	1.001868	124.52	1.001396	125.49
0.577544	10.0022	1.002480	110.93	1.002010	111.34
1.50303	10.0022	1.002557	112.95	1.002086	113.52
3.02047	10.0022	1.002676	115.71	1.002205	116.10
5.05020	10.0022	1.002830	117.97	1.002359	118.21
7.02760	10.0022	1.002971	120.07	1.002500	120.25
		**298.15 K**		**300.15 K**	
0.476397	0.500080	0.997364	121.39	0.996813	121.17
1.51641	0.500080	0.997439	123.67	0.996890	122.41
3.03180	0.500080	0.997547	124.85	0.997000	123.60
4.97062	0.500080	0.997679	126.46	0.997137	124.83
7.03538	0.500080	0.997815	127.97	0.997279	125.88
0.496817	2.50144	0.998180	124.82	0.997644	125.50
1.47082	2.50144	0.998250	125.34	0.997713	125.93
3.00163	2.50144	0.998358	126.00	0.997821	126.34
5.05041	2.50144	0.998501	126.56	0.997964	126.89
6.92625	2.50144	0.998631	126.99	0.998093	127.24
0.496884	4.96387	0.999221	120.01	0.998689	120.84
1.47060	4.96387	0.999295	120.60	0.998762	121.24
3.08245	4.96387	0.999416	121.33	0.998883	121.81
5.02990	4.96387	0.999561	121.90	0.999028	122.26
7.10753	4.96387	0.999714	122.32	0.999180	122.70
0.516978	7.48862	1.000432	118.06	0.999890	118.75
1.55137	7.48862	1.000511	120.26	0.999968	120.88

$10^3\ m_{L\text{-dopa}}$ /(mol Kg^{-1})	$10^3\ m_{HP\text{-}\beta\text{-}CD}$ /(mol Kg^{-1})	ρ /(g cm^{-3})	V_ϕ /(cm^3 mol^{-1})	ρ /(g cm^{-3})	V_ϕ /(cm^3 mol^{-1})
3.03198	7.48862	1.000618	122.44	1.000074	123.22
4.99891	7.48862	1.000755	124.41	1.000210	125.16
7.11888	7.48862	1.000897	126.10	1.000351	126.71
0.577544	10.0022	1.001510	111.73	1.000965	112.13
1.50303	10.0022	1.001585	114.08	1.001040	114.57
3.02047	10.0022	1.001703	116.71	1.001157	117.25
5.05020	10.0022	1.001854	119.10	1.001307	119.66
7.02760	10.0022	1.001995	120.95	1.001445	121.79
		**302.15 K**		**304.15 K**	
0.476397	0.500080	0.996248	121.35	0.995647	121.59
1.51641	0.500080	0.996325	122.57	0.995724	122.93
3.03180	0.500080	0.996435	123.76	0.995833	124.19
4.97062	0.500080	0.996572	124.90	0.995969	125.37
7.03538	0.500080	0.996714	125.96	0.996112	126.17
0.496817	2.50144	0.997077	125.79	0.996475	126.01
1.47082	2.50144	0.997146	126.26	0.996544	126.49
3.00163	2.50144	0.997254	126.68	0.996651	127.05
5.05041	2.50144	0.997396	127.11	0.996792	127.54
6.92625	2.50144	0.997526	127.41	0.996921	127.88
0.496884	4.96387	0.998119	121.40	0.997515	121.93
1.47060	4.96387	0.998193	121.86	0.997588	122.39
3.08245	4.96387	0.998313	122.35	0.997707	122.97
5.02990	4.96387	0.998456	122.90	0.997849	123.40
7.10753	4.96387	0.998608	123.16	0.997999	123.87
0.516978	7.48862	0.999318	119.39	0.998711	120.01
1.55137	7.48862	0.999396	121.56	0.998788	122.18
3.03198	7.48862	0.999501	123.70	0.998893	124.26
4.99891	7.48862	0.999635	125.80	0.999026	126.35
7.11888	7.48862	0.999775	127.37	0.999165	127.85
0.577544	10.0022	1.000390	112.85	0.999780	113.73
1.50303	10.0022	1.000465	115.20	0.999854	115.88
3.02047	10.0022	1.000581	117.79	0.999970	118.48
5.05020	10.0022	1.000729	120.36	1.000117	120.88
7.02760	10.0022	1.000865	122.59	1.000255	122.69
		**306.15 K**		**308.15 K**	
0.476397	0.500080	0.995012	121.64	0.994340	121.92
1.51641	0.500080	0.995088	123.17	0.994417	123.48
3.03180	0.500080	0.995197	124.46	0.994525	124.83
4.97062	0.500080	0.995332	125.66	0.994660	125.97
7.03538	0.500080	0.995472	126.82	0.994800	126.99
0.496817	2.50144	0.995837	126.24	0.995164	126.48
1.47082	2.50144	0.995906	126.71	0.995233	126.97
3.00163	2.50144	0.996013	127.17	0.995339	127.39
5.05041	2.50144	0.996154	127.69	0.995480	127.96
6.92625	2.50144	0.996282	128.07	0.995608	128.28
0.496884	4.96387	0.996875	122.47	0.996200	122.97
1.47060	4.96387	0.996948	122.90	0.996272	123.34
3.08245	4.96387	0.997066	123.37	0.996390	123.85

$10^3\ m_{L-dopa}$ /(mol Kg^{-1})	$10^3\ m_{HP-β-CD}$ /(mol Kg^{-1})	ρ /(g cm^{-3})	V_ϕ /(cm^3 mol^{-1})	ρ /(g cm^{-3})	V_ϕ /(cm^3 mol^{-1})
5.02990	4.96387	0.997208	123.85	0.996531	124.26
7.10753	4.96387	0.997357	124.27	0.996680	124.64
0.516978	7.48862	0.998069	120.63	0.997391	121.23
1.55137	7.48862	0.998145	122.79	0.997466	123.34
3.03198	7.48862	0.998249	124.86	0.997569	125.35
4.99891	7.48862	0.998381	126.83	0.997702	127.13
7.11888	7.48862	0.998520	128.27	0.997839	128.77
0.577544	10.0022	0.999135	114.25	0.998456	115.11
1.50303	10.0022	0.999209	116.63	0.998528	117.44
3.02047	10.0022	0.999323	119.19	0.998642	119.78
5.05020	10.0022	0.999470	121.42	0.998787	122.19
7.02760	10.0022	0.999606	123.37	0.998920	124.36
		**310.15 K**		**312.15 K**	
0.476397	0.500080	0.993612	122.20	0.992892	122.56
1.51641	0.500080	0.993688	123.86	0.992967	124.33
3.03180	0.500080	0.993796	125.21	0.993074	125.63
4.97062	0.500080	0.993927	126.96	0.993206	127.23
7.03538	0.500080	0.994065	128.04	0.993342	128.47
0.496817	2.50144	0.994451	127.05	0.993712	127.50
1.47082	2.50144	0.994519	127.47	0.993780	127.85
3.00163	2.50144	0.994625	127.86	0.993885	128.20
5.05041	2.50144	0.994766	128.25	0.994025	128.58
6.92625	2.50144	0.994893	128.59	0.994153	128.80
0.496884	4.96387	0.995492	123.55	0.994745	124.03
1.47060	4.96387	0.995564	123.97	0.994816	124.36
3.08245	4.96387	0.995681	124.40	0.994932	124.84
5.02990	4.96387	0.995820	124.91	0.995071	125.36
7.10753	4.96387	0.995968	125.26	0.995218	125.73
0.516978	7.48862	0.996671	121.77	0.995928	122.40
1.55137	7.48862	0.996747	123.75	0.996003	124.30
3.03198	7.48862	0.996849	125.88	0.996105	126.35
4.99891	7.48862	0.996981	127.59	0.996235	128.30
7.11888	7.48862	0.997116	129.39	0.996370	129.90
0.577544	10.0022	0.997736	115.80	0.996991	116.79
1.50303	10.0022	0.997808	118.26	0.997063	118.93
3.02047	10.0022	0.997921	120.54	0.997174	121.39
5.05020	10.0022	0.998065	122.85	0.997316	123.78
7.02760	10.0022	0.998197	125.00	0.997447	125.82

Table S8. Partial molar volumes at infinitesimal concentration, $V_{\phi}^{0}= V_{2}^{0}$, and partial molar volumes of transfer, ΔV_{ϕ}^{0}, for aqueous solutions of L-dopa in the presence of β-CD, at temperatures from 294.15 K to 312.15 K.

$10^3\ m_{\beta\text{-CD}}$ /(mol Kg^{-1})	V_{ϕ}^{0} /(cm^3 mol^{-1}) [(a)]	ΔV_{ϕ}^{0} /(cm^3 mol^{-1})	V_{ϕ}^{0} /(cm^3 mol^{-1}) [(a)]	ΔV_{ϕ}^{0} /(cm^3 mol^{-1})
	**294.15 K**		**296.15 K**	
0	122.21		122.48	
0.499980	111.61	-10.6	115.72	-6.76
2.479425	113.17	-9.04	120.6	-1.88
4.993389	117.42	-4.79	122.73	0.25
7.410455	128.05	5.84	132.7	10.22
10.000811	137.12	14.91	138.37	15.89
	**298.15 K**		**300.15 K**	
0	123.01		123.49	
0.499980	123.55	0.54	127.42	3.93
2.479425	126.27	3.26	128.86	5.37
4.993389	128.17	5.16	133.25	9.76
7.410455	135.79	12.78	139.46	15.97
10.000811	141.00	17.99	143.77	20.28
	**302.15 K**		**304.15 K**	
0	124.10		124.48	
0.499980	130.82	6.72	133.70	9.22
2.479425	132.22	8.12	136.11	11.63
4.993389	137.24	13.14	139.99	15.51
7.410455	141.27	17.17	143.34	18.86
10.000811	146.3	22.2	147.45	22.97
	**306.15 K**		**308.15 K**	
0	124.96		125.58	
0.499980	139.59	14.63	143.87	18.29
2.479425	140.74	15.78	145.55	19.97
4.993389	142.44	17.48	147.01	21.43
7.410455	145.94	20.98	148.45	22.87
10.000811	148.35	23.39	150.19	24.61
	**310.15 K**		**312.15 K**	
0	126.02		126.32	
0.499980	147.53	21.51	150.73	24.41
2.479425	148.19	22.17	152.28	25.96
4.993389	149.10	23.08	152.49	26.17
7.410455	151.15	25.13	153.71	27.39
10.000811	151.94	25.92	154.15	27.83

Table S9. Partial molar volumes at infinitesimal concentration, $V_{\phi}^{0}= V_{2}^{0}$, and partial molar volumes of transfer, ΔV_{ϕ}^{0}, for aqueous solutions of L-dopa in the presence of HP-β-CD, at temperatures from 294.15 K to 312.15 K.

10^3 $m_{HP\text{-}\beta\text{-}CD}$ /(mol Kg-1)	V_{ϕ}^{0} /(cm^3 mol^{-1}) (a)	ΔV_{ϕ}^{0} /(cm^3 mol^{-1})	V_{ϕ}^{0} /(cm^3 mol^{-1}) (a)	ΔV_{ϕ}^{0} /(cm^3 mol^{-1})
	**294.15 K**		**296.15 K**	
0.000000	122.21		122.48	
0.500080	118.32	-3.89	118.92	-3.56
2.50144	122.66	0.45	123.11	0.63
4.96387	116.11	-6.10	118.23	-4.25
7.48862	113.95	-8.26	114.53	-7.95
10.0022	107.16	-15.05	107.81	-14.67
	**298.15 K**		**300.15 K**	
0.000000	123.01		123.49	
0.500080	119.33	-3.68	119.48	-4.01
2.50144	124.00	0.99	124.83	1.34
4.96387	119.17	-3.84	120.13	-3.36
7.48862	115.12	-7.89	115.83	-7.66
10.0022	108.08	-14.93	108.32	-15.17
	**302.15 K**		**304.15 K**	
0.000000	124.10		124.48	
0.500080	119.70	-4.40	120.01	-4.47
2.50144	125.22	1.12	125.32	0.84
4.96387	120.75	-3.35	121.22	-3.26
7.48862	116.44	-7.66	117.86	-6.62
10.0022	108.92	-15.18	110.71	-13.77
	**306.15 K**		**308.15 K**	
0.000000	124.96		125.58	
0.500080	119.88	-5.08	120.25	-5.33
2.50144	125.55	0.59	125.81	0.23
4.96387	121.79	-3.17	122.33	-3.25
7.48862	117.86	-7.10	118.51	-7.07
10.0022	110.71	-14.25	111.44	-14.14
	**310.15 K**		**312.15 K**	
0.000000	126.02		126.32	
0.500080	120.12	-5.90	120.53	-5.79
2.50144	126.50	0.48	127.03	0.710
4.96387	122.90	-3.12	123.34	-2.98
7.48862	118.96	-7.06	119.55	-6.77
10.0022	112.24	-13.78	113.12	-13.20

Table S10. Partial molar expansibility at infinite dilution, E_2^0, of L-dopa in presence, of β-CD at different concentrations.

$10^3\, m_{\beta\text{-CD}}$ /(mol Kg^{-1})	E_2^0 /(cm^3 mol^{-1} K^{-1})				
	**294.15 K**	**296.15 K**	**298.15 K**	**300.15 K**	**302.15 K**
0.499980	2.619	2.520	2.422	2.324	2.225
2.479425	2.470	2.381	2.291	2.201	2.112
4.993389	2.713	2.531	2.349	2.167	1.985
7.410455	1.715	1.637	1.559	1.481	1.403
10.00081	1.152	1.097	1.043	0.989	0.934
	**304.15 K**	**306.15 K**	**308.15 K**	**310.15 K**	**312.15 K**
0.499980	2.127	2.028	1.930	1.832	1.733
2.479425	2.022	1.933	1.843	1.753	1.664
4.993389	1.803	1.621	1.439	1.257	1.075
7.410455	1.325	1.247	1.169	1.091	1.013
10.00081	0.880	0.825	0.771	0.717	0.662

Table S11. Partial molar expansibility at infinite dilution, E_2^0, of L-dopa in presence, of HP-β-CD at different concentrations.

10^3 $m_{HP\text{-}\beta\text{-CD}}$ /(mol Kg^{-1})	E_2^0 /(cm^3 mol^{-1} K^{-1})				
	**294.15 K**	**296.15 K**	**298.15 K**	**300.15 K**	**302.15 K**
0.500080	0.142	0.134	0.126	0.118	0.110
2.50144	0.350	0.328	0.307	0.285	0.263
4.96387	0.649	0.588	0.527	0.466	0.405
7.48862	0.395	0.380	0.365	0.350	0.336
10.0022	0.366	0.344	0.323	0.302	0.281
	**304.15 K**	**306.15 K**	**308.15 K**	**310.15 K**	**312.15 K**
0.500080	0.102	0.094	0.086	0.078	0.070
2.50144	0.242	0.220	0.199	0.177	0.155
4.96387	0.343	0.282	0.221	0.160	0.099
7.48862	0.321	0.306	0.291	0.276	0.262
10.0022	0.260	0.238	0.217	0.196	0.175

Table. S12. Hepler constant to the system L-dopa–β–CD and L-dopa–HP-β–CD

10^3 $m_{\beta\text{-CD}}$ /(mol Kg^{-1})	$\left(\frac{\partial E_2^0}{\partial T}\right)$ / (cm^3 mol^{-1} K^{-2})	10^3 $m_{HP\text{-}\beta\text{-CD}}$ /(mol Kg^{-1})	$\left(\frac{\partial E_2^0}{\partial T}\right)$ / (cm^3 mol^{-1} K^{-2})
0.499980	-0.049	0.500080	-0.004
2.479425	-0.045	2.50144	-0.011
4.993389	-0.091	4.96387	-0.031
7.410455	-0.039	7.48862	-0.007
10.00081	-0.027	10.0022	-0.011

Table. S13. Estimated thermodynamic properties for the complexation process of L-dopa with β-CD, at temperatures from 294.15 K to 312.15 K

T / K	***K*** /(kJ·mol^{-1})	***ΔG⁰*** /(kJ·mol^{-1})	***ΔS⁰*** /(kJ·mol^{-1})	***ΔH⁰*** /(kJ·mol^{-1})
294.15	1791.51	-18.31	-0.13	-57.15
296.15	1284.16	-17.62		
298.15	1319.74	-17.80		
300.15	618.32	-16.03		
302.15	657.71	-16.29		
304.15	677.38	-16.47		
306.15	744.21	-16.82		
308.15	499.7	-15.91		
310.15	450.84	-15.75		
312.15	421.02	-15.67		

Table S14. Estimated thermodynamic properties for the complexation process of L-dopa with HP-β-CD, at temperatures from 294.15 K to 312.15 K

T / K	*K* /(kJ·mol^{-1})	*ΔG^0* /(kJ·mol^{-1})	*ΔS^0* /(kJ·mol^{-1})	*ΔH^0* /(kJ·mol^{-1})
294.15	442.5	-14.89	0.14	26.73
296.15	448.4	-15.03		
298.15	546.3	-15.62		
300.15	625.5	-16.06		
302.15	629.9	-16.18		
304.15	547.1	-15.93		
306.15	613.4	-16.33		
308.15	649.1	-16.58		
310.15	796.4	-17.22		
312.15	953.8	-17.80		

Table S15. Viscosity of the solutions of L-dopa in presence of the mixed solvent {water + β-CD}, a 298.15 K and 310.15 K.

$10^3\ m_{\text{L-Dopa}}$ /(mol Kg^{-1})	$10^3\ m_{\beta\text{-CD}}$ /(mol Kg^{-1})	η /cP 298.15 K	η /cP 310.15 K
0.520000	0.499980	0.8916	0.6934
1.550000	0.499980	0.8926	0.6937
3.041948	0.499980	0.8974	0.6968
5.122107	0.499980	0.9025	0.7013
7.165244	0.499980	0.9077	0.7062
0.497001	2.479425	0.8955	0.6968
1.521035	2.479425	0.8969	0.6981
3.053917	2.479425	0.8994	0.6993
5.021402	2.479425	0.9018	0.7015
7.456409	2.479425	0.9052	0.7042
0.527446	4.993389	0.9011	0.7045
1.651840	4.993389	0.9022	0.7058
3.063624	4.993389	0.9039	0.7073
5.041076	4.993389	0.9058	0.7087
7.014787	4.993389	0.9078	0.7100
0.537525	7.410455	0.9059	0.7099
1.601455	7.410455	0.9080	0.7110
3.040403	7.410455	0.9101	0.7120
4.959436	7.410455	0.9120	0.7136
6.942657	7.410455	0.9149	0.7156
0.527430	10.00081	0.9100	0.7135
1.490858	10.00081	0.9120	0.7145
2.952377	10.00081	0.9137	0.7161
4.946662	10.00081	0.9160	0.7176
6.957784	10.00081	0.9188	0.7193

Table S16. Viscosity of the solutions of L-dopa in presence of the mixed solvent {water + HP-β-CD}, a 298.15 K and 310.15 K.

10^3 $m_{\text{L-Dopa}}$ /(mol Kg^{-1})	10^3 $m_{\text{HP-β-CD}}$ /(mol Kg^{-1})	η /cP 298.15 K	η /cP 310.15 K
0.476397	0.500080	0.8920	0.6936
1.51641	0.500080	0.8935	0.6948
3.03180	0.500080	0.8959	0.6966
4.97062	0.500080	0.8984	0.6985
7.03538	0.500080	0.9013	0.7014
0.496817	2.50144	0.8988	0.6990
1.47082	2.50144	0.9000	0.6988
3.00163	2.50144	0.9021	0.7003
5.05041	2.50144	0.9049	0.7022
6.92625	2.50144	0.9070	0.7047
0.496884	4.96387	0.9017	0.7035
1.47060	4.96387	0.9040	0.7037
3.08245	4.96387	0.9069	0.7054
5.02990	4.96387	0.9100	0.7081
7.10753	4.96387	0.9154	0.7116
0.516978	7.48862	0.9101	0.7168
1.55137	7.48862	0.9125	0.7176
3.03198	7.48862	0.9149	0.7188
4.99891	7.48862	0.9190	0.7204
7.11888	7.48862	0.9234	0.7220
0.577544	10.0022	0.9184	0.7227
1.50303	10.0022	0.9199	0.7243
3.02047	10.0022	0.9255	0.7265
5.05020	10.0022	0.9339	0.7301
7.02760	10.0022	0.9386	0.7322

Table S17 Coefficients A and B of Jones-Dole equation and viscosity *B*-coefficients of transfer, ΔB, for aqueous solutions of L-dopa in the presence of β-CD, at 298.15 K and 310.15 K.

10^3 $m_{β\text{-}CD}$ /(mol·dm^{-3})	*A* /(dm$^{3/2}$·mol$^{-1/2}$)	*B* /(dm^3·mol^{-1})	*ΔB* /(dm^3·mol^{-1})	*A* /(dm$^{3/2}$·mol$^{-1/2}$)	*B* /(dm^3·mol^{-1})	*ΔB* /(dm^3·mol^{-1})
	**298.15 K**			**310.15 K**		
0.000000		0.52			0.80	
0.499980	-0.08	3.51	2.99	-0.09	3.78	2.98
2.479425	0.02	1.39	0.87	-0.02	1.71	0.91
4.993389	0.06	0.59	0.07	0.12	0.12	-0.68
7.410455	0.13	0.34	-0.18	0.14	0.01	-0.79
10.00081	0.11	0.44	-0.08	0.04	0.90	0.10

Table S18 . Coefficients A and B of Jones-Dole equation and viscosity *B*-coefficients of transfer, ΔB, for aqueous solutions of L-dopa in the presence of HP-β-CD, at 298.15 K and 310.15 K.

10^3 $m_{HP\text{-}\beta\text{-}CD}$ /(moldm^{-3})	*A* /(dm$^{3/2}$·mol$^{-1/2}$)	*B* /(dm^3·mol^{-1})	*ΔB* /(dm^3·mol^{-1})	*A* /(dm$^{3/2}$·mol$^{-1/2}$)	*B* /(dm^3·mol^{-1})	*ΔB* /(dm^3·mol^{-1})
		**298.15 K**			**310.15 K**	
0.000000		0.52			0.80	
0.500080	0.00	1.55	1.03	-0.02	1.84	1.04
2.50144	0.13	0.26	-0.26	-0.16	2.83	2.03
4.96387	-0.42	5.52	5.00	0.02	2.01	1.21
7.48862	-0.11	2.04	1.52	0.18	0.65	-0.15
10.0022	-0.29	4.54	4.02	0.25	1.47	0.67
